# Ceramic-to-metal bonding using rare-earth containing Sn–Bi solder

**DOI:** 10.1007/s10854-024-12176-5

**Published:** 2024-02-21

**Authors:** Tianshi Feng, Bhabana Pati, Ka Man Chung, Yu Pei, Renkun Chen

**Affiliations:** 1 Department of Mechanical and Aerospace Engineering, University of California San Diego, 9500 Gilman Drive, MC 0411, La Jolla, CA 92130-0411 USA; 2 Physical Sciences, Inc, 20 New England Business Center Drive, Andover, MA 01810 USA; 3 Program in Materials Science and Engineering, University of California San Diego, 9500 Gilman Drive, MC 0418, La Jolla, CA 92093-0418 USA

## Abstract

With the increasing miniaturization and power of optoelectronic devices, direct bonding of optical substrates like semiconductors and ceramics to metal heat sinks using low melting-point solder has gained significant interest. In this study, we demonstrated the bonding of glass to copper using Sn-58 wt% Bi solder (SB solder) doped with a small amount of rare earth (RE) elements. The RE elements act as active agents that facilitate the bonding to glasses without glass metallization. By optimizing the bonding parameters, such as reflow temperature and time, and employing an inert gas atmosphere to prevent solder or RE oxidation, we successfully achieved the highest shear strength in glass-copper solder joints using SB-RE solder, without the need for ultrasonic-assisted soldering (UAS). These results demonstrate the potential of using RE-containing solder for bonding unmetallized glass and ceramics in optoelectronic devices with metals at low soldering temperatures (< 200 °C). Furthermore, analysis of the shear strength and failure morphology of solder joints revealed only small degradation, primarily originating from the bulk solder region rather than the solder-glass interface, after both thermal aging (100 h) and cycling tests (100 cycles). The establishment of low-melting point RE-containing solders opens the possibility of direct jointing ceramic optoelectronic substrates to metal heat sinks for more efficient heat dissipation. In the meantime, our work also suggests that further optimization studies are necessary to explore its performance under more extreme working conditions.

## Introduction

As modern electronic and optoelectronic devices continue to develop, bonding processes on ceramic, oxide, and semiconductor substrates are increasingly utilized in package assembly. However, conventional solders used in industries have trouble wetting at semiconductor and optical substrates made of ceramics such as SiO_2_, sapphire, Nd:YAG (neodymium-doped yttrium aluminum garnet; Nd:Y_3_Al_5_O_12_), and BBO (Beta Barium Borate). To achieve bonding on these substrates, surface metallization has been commonly employed with commercially available solder options, including Sn-based Pb-free solders with different melting points [1–4]. However, the bonding strength of the metallization layer and limitations of the substrate sizes and geometries pose challenges when using common solder with subsequent thin metal layer deposition [5]. Thus, there is a need for a direct and robust bonding solution on these non-wettable substrates.

To address this issue, researchers have investigated the use of “universal solders,” which are traditional solders doped with trace amounts of rare earth (RE) or other active elements like titanium (Ti) for improving solder joint qualities on ceramics without the need for metallization [6–8]. The active elements exhibit a stronger affinity to oxygen, facilitating direct and strong bonding between the solders and traditionally non-wettable, oxide-based optical and semiconductor substrates [9–13].

For temperature-sensitive optoelectronic devices, the maximum soldering temperature is often limited, and bonding typically needs to be carried out at a reflow temperature above the melting point of the solder. Therefore, low-temperature Pb-free solders with the melting point ranging from 118 to 170 °C and reflow temperatures between 130 and 200 °C have garnered significant attention due to the benefits of reducing energy costs during soldering and improving the lifetime of electronic devices [14]. An example of a common low-temperature solder is eutectic Sn-58 wt% Bi (SB) with a melting pointof ~ 140 °C [15]. The SB solder has achieved solder joint strength exceeding 30 MPa with a 200 °C reflow temperature when electroplated onto copper substrates [16]. Typically, bonding SB solders to metal surfaces yields high shear strength on the order of 30–50 MPa [16]. However, when bonding is attempted without metallization on oxide or ceramic substrates and using active elements such as RE or Ti, soldering processes often result in moderate bonding strength, typically around 1 MPa [5], unless an ultrasonic-assisted soldering process is employed, which can increase the bonding strength to 2–11 MPa [17, 18]. However, as far as we know, no attempt has been made to achieve higher bonding strength with RE- or Ti- containing SB solders.

Yonekura et al. [19] studied Sn–Zn–Sb solder bonding (~ 350 °C soldering reflow temperature with > 200 °C melting point) to glass and found that the solder could only wet the glass when using ultrasonic assistance. They achieved bonding shear strength ranging from 1.03 to 5.93 MPa, with an average value of 2.90 Mpa. Additionally, they observed little growth of intermetallic compounds (IMCs) at the solder-glass interfaces. Table 1 provides a summary of the state-of-the-art solder joint shear strength using different types of active solders and various soldering processes. Notably, there is no systematic study on the long-term thermal cycling and aging stability of the active solder joints.

**Table 1:** Summary of state-of-art solder joints for bonding of ceramics, oxides, and semiconductors using active solder (UAS: ultrasonic-assisted soldering)

Solder [ref]	Melting point [^o^C]	Substrates	Bonding Methods	Max Shear Strength (MPa)
S-Bond 140 M1 (SB with Ti) [17]	138	High-purity fused silica	UAS	11.5 ± 5.8
S-Bond 140 M1 [18]	138	Glass slides	UAS	2 ± 1 (bulk) 2.5 ± 2 (interfacial)
Sn–58Bi–4RE [5]	140	Borosilicate glass	Manual	> 0.55
Sn–Zn–Sb [19]	> 200	Glass slides	UAS	1.03–5.93 (avg: 2.90)
92Sn3Ag3Ti [9]	220–235	Al_2_O_3_/Al alloy	UAS	30–65
Sn–3.5Ag–4Ti [22]	221–232	SiO_2_	Manual	11.15 (1 min) 14.1 (15 min) 16.37 (30 min) 17.91 (1 h)
Sn–3.5Ag–4Ti [27]	221–232	Si	Manual	3.67
Sn–3.5Ag–5Ce [13]	222	Si	Manual	14 (250 °C soldering temperature)

**Fig. 1:**
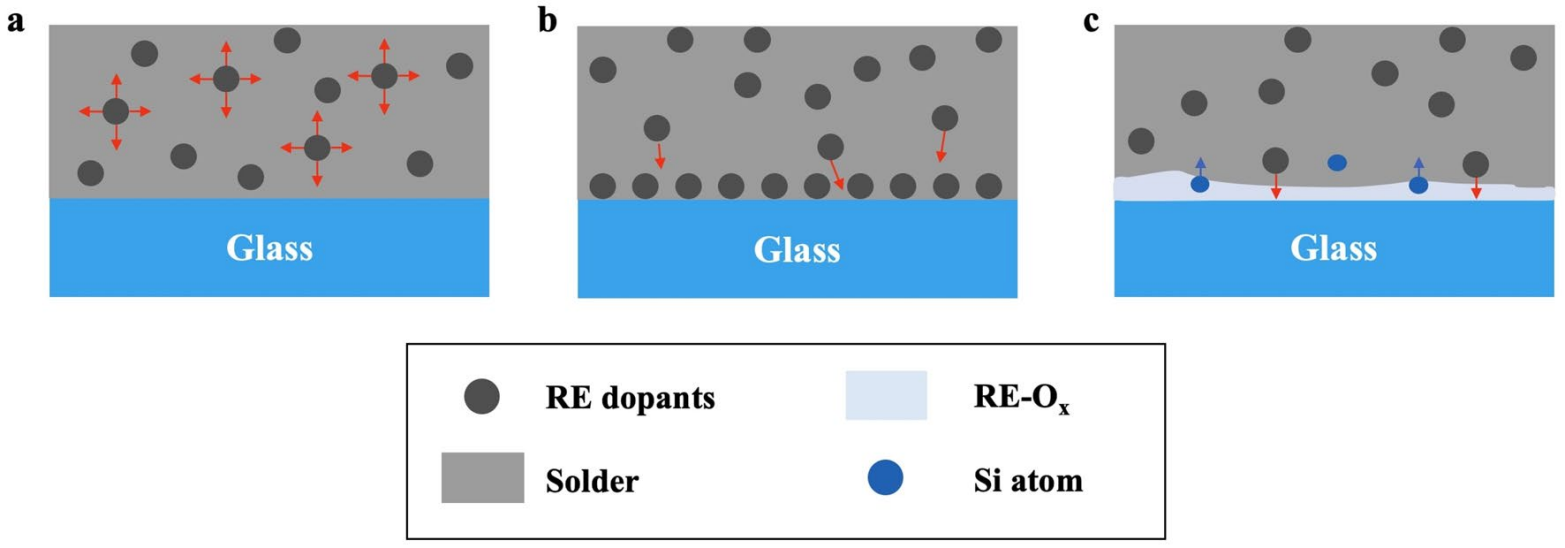
Schematics of active solder bonding to glass (oxide surface). **a** Doped RE elements free diffusion in the solder matrix melt at the beginning of the soldering process. **b** The RE elements from the solder diffuse toward the glass interface. **c **Segregated RE elements at the interface of glass react with the SiO_2,_ creating a thin interfacial layer with strong chemical bonds

In this work, we introduced inexpensive mischmetals as the RE sources into the SB solder to achieve direct and robust bonding between bare glass and copper. This solder, referred to as “universal solder”, has been previously investigated by our team [5]. In our bonding process, we used bare copper and glass without any metallization or surface treatment. Only gentle cleaning using organic solvents was required. As illustrated in Fig. 1, the active solder facilitates the direct bonding to glass substrates through the following process [17, 20–22]: (i) At the start of the soldering process, RE elements freely diffuse within the molten solder; (ii) Upon reaching the glass interface, the RE elements form covalent bonds with exposed oxygen atoms on the glass surface, allowing the absorption and accumulation of RE elements from the solder; (3) As the soldering time increases, more RE elements diffuse towards the glass interface, initiating a reaction between the RE elements and SiO_2_. This reaction ultimately forms a thin interfacial layer, resulting in strong bonding between the glass and the solder. Table 2 provides a summary of the heat of formation of several oxide compounds [17]. Due to the lower heat of formation of active elements (RE, Ti) with oxygen compared to that of Si and O_2_ forming SiO_2_, it is energetically more favorable to form RE/Ti–O compounds, facilitating the reaction between the active elements and the SiO_2_ substrates for a strong bonding. In this study, we optimized the bonding process by embedding RE particles into the solder matrix to achieve a more uniform RE distribution, creating uniform solder sheets for bonding. We systematically investigated the effects of RE concentration, reflow temperature, and reflow time on the shear strength of the solder joint. We demonstrate that our best soldering joints exhibited higher shear strength compared to the state-of-the-art SB solders. We also conducted long-term thermal cycling and aging stability tests of the solder joints. The growth of intermetallic compounds (IMCs) within the solder joints during long-term, high-temperature (> 160 °C) operation is a common concern in electronic and optoelectronic devices [15]. Additionally, solder joints in these devices may be subjected to overheating or other extreme working conditions, which can shorten their lifetime [23–26]. We evaluated solder joint degradation through shear testing after various post-thermal processes, observing different degrees of degradation in solder strength. Overall, our studied solder joints maintained promising mechanical strength after thermal aging and cycling tests. These results suggest that our universal solders show promise for direct bonding of optoelectronic ceramic substrates with metals. Further investigations are also suggested to enhance the thermal stability of solder joints under more extreme working conditions.

**Table 2:** Heat formation of selected oxides; a more negative value indicates more energetically favorable for the formation of the oxide [17]

Material Class	Compound	Heat of Formation (kJ/mol)
Rare Earth (Lanthanide) Oxides	Er_2_O_3_	− 1898
	Lu_2_O_3_	− 1878
	Ce_2_O_3_	− 1896
	Al_2_O_3_	− 1675
	TiO_2_	− 944
Quartz	SiO_2_	− 911
Silica (fused quartz)	SiO_2_	− 903.5

## Experimental methods

### Fabrication of RE-containing solder sheets

**Fig. 2:**
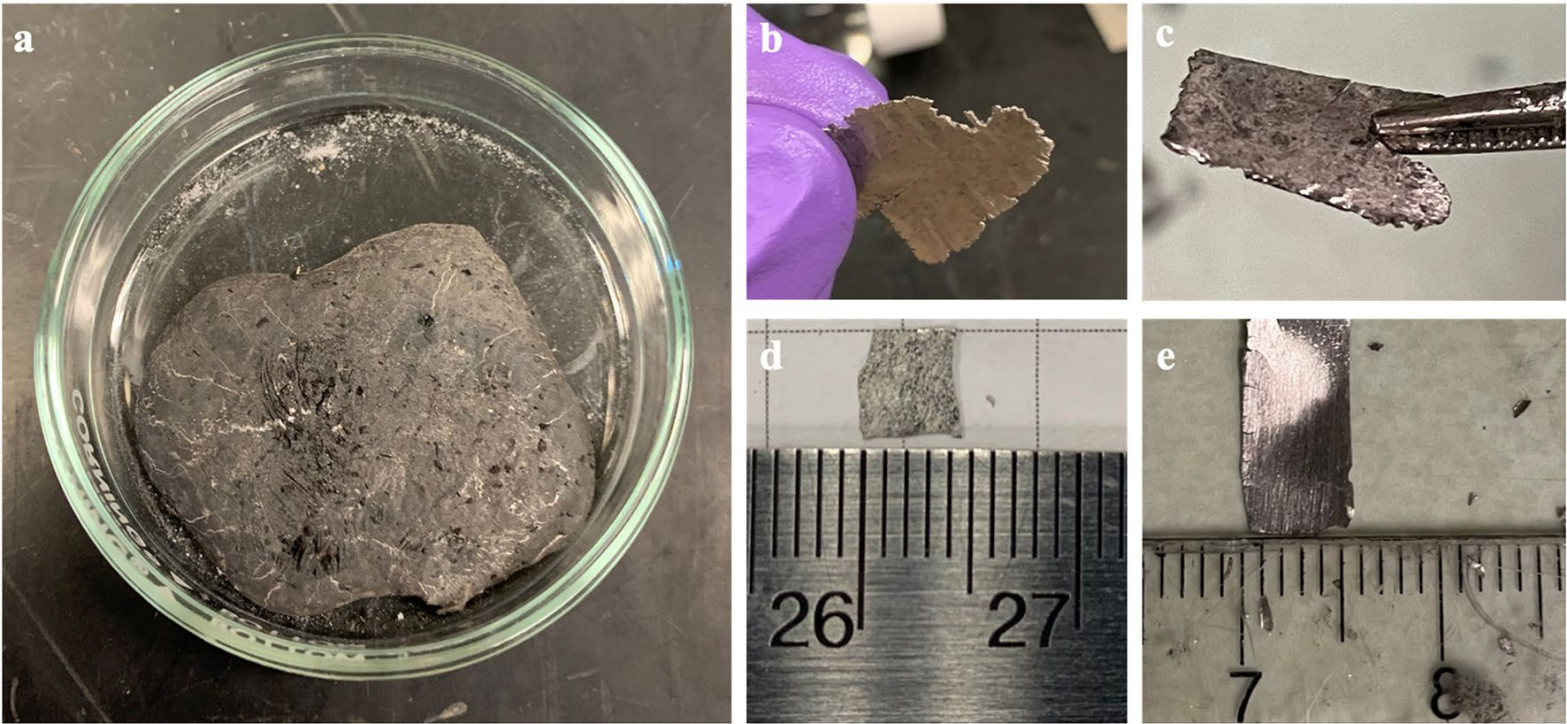
As-fabricated SnBi-xRE solder. **a** Bulk universal solder after mixing RE particles into the molten Sn58Bi solder matrix; **b** Universal solder sheets after rolling; **c–e** Difference sizes and shapes after cutting the solder sheets

Sn-58 wt% Bi (SB) solder bars were purchased from McMaster-Carr (Stock No. 8921k16), and RE mischmetal containing Ce:La (75:25 wt%) was obtained from Alfa Aesar (stock No. 45589-36). Both materials were used as received without further treatment. Initially, fine particles of the RE were prepared by ball milling the mischmetal for four hours. Prior to ball milling, the RE shots were loaded into a small stainless-steel container inside an Ar environment glove box with an oxygen concentration less than 0.5 ppm to prevent oxidation. The container was then sealed, taken out of the glovebox, and loaded into a ball milling machine (Mixer/Mill 8000 M, SPEX SamplePrep). After ball milling, the RE particles in the container were transferred back to the glovebox for mixing. In the meantime, molten SB solder was prepared by melting it in a glass petri dish on a hot plate inside the Ar glove box. The RE particles with predetermined concentrations (ranging from 2 to 8 wt% in this study) were then added to the molten SB solder and mixed. During the mixing process, a magnetic stirring bar in the petri dish continuously stirred the molten SB and RE particles for approximately 20 min to ensure uniform mixing. After mixing, the solder was cooled to room temperature and removed from the glove box. The solder was then rolled into thin sheets with a thickness of around 50 μm using a rolling machine (Compact Economy Rolling Mill, Contenti, # 190–889). Smaller sheets with suitable sizes (typically around 5 × 5 mm^2^) were cut from the larger sheets for the soldering experiments. Figure 2 shows the as-fabricated universal solders in different shapes (bulk solder after mixing, thin sheets after rolling, and specific sizes after cutting of solder thin sheets). At this point, since the RE particles were embedded in the solder matrix, they were protected from oxidation and could be safely stored in air.

### Soldering process

Since RE is highly sensitive to oxygen, we conducted the soldering process in an Ar-filled glovebox to avoid deactivation of the RE when heated. For comparison, the soldering processes were performed with three different reflow temperatures (170 °C, 200 °C, and 240 °C) and three different reflow times (5 min, 10 min, and 30 min). We employed the same manual soldering procedure on all the studied joints using a hot plate as the heating source. The solder sheet was applied to each substrate (Glass and Cu) first, and we spread the molten solder until the substrates were fully wetted at the desired reflow temperature. Next, we sandwiched the two wet substrates together with a certain weight (~ 1 kg) on top of them for a while. The solder joints were naturally cooled down to room temperature eventually.

### Shear test

**Fig. 3:**
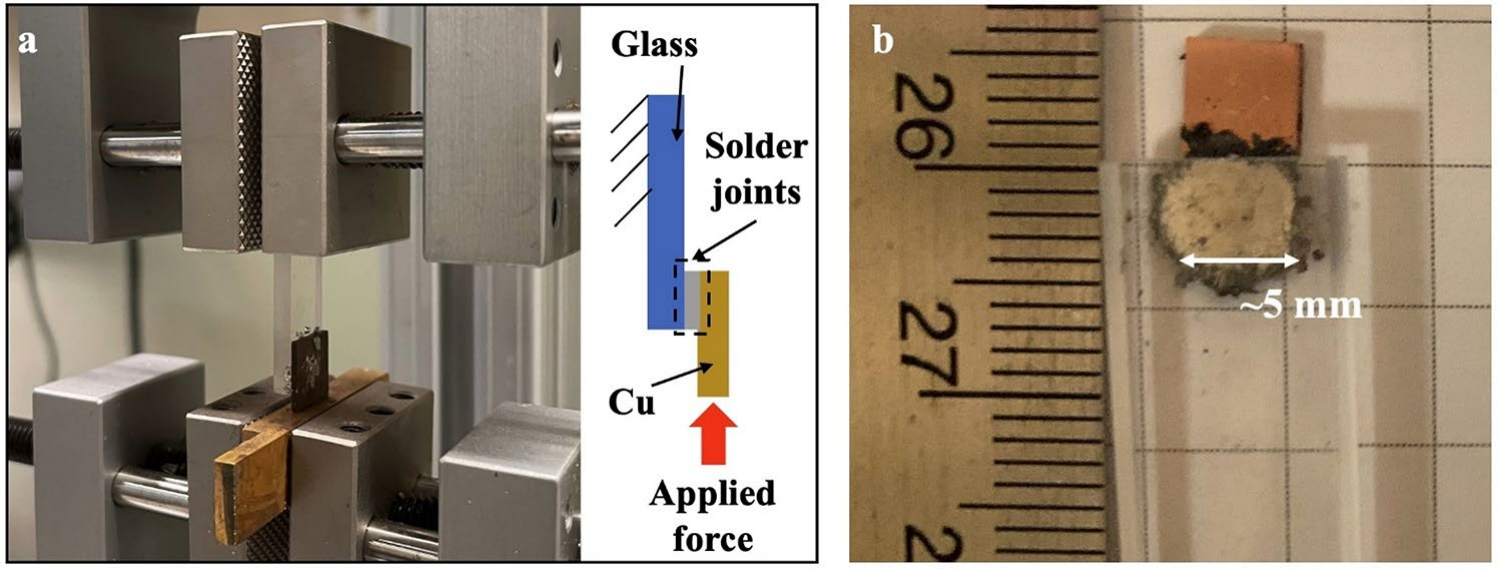
**a** Photograph and schematics of the shear test setup on Cu/SnBi-xRE/SiO_2_ solder joints; **b** Image of as-bonded Cu/SnBi-xRE/SiO_2_ solder joints with a bonding area of approximately 5 × 5 mm^2^

The shear tests on the solder joints were performed using a Universal Tensile Machine (TSA750, MARK-10. Co). The glass substrates were fixed during the shear test. An accumulated force, monitored by a Force Gauge (M3-200, MARK-10. Co), was applied to the copper substrates until the solder joints failed (Fig. 3). The maximum shear strength was calculated by dividing the maximum force by the bonding area. We measured the bonding area for each solder joint before the shear test, typically controlling it to be around 5 × 5 mm^2^ (Fig. 3). All the shear tests were conducted on 6–8 solder joint samples for each bonding condition to obtain an average value and a standard deviation of the specific condition.

### Thermal aging test

The growth of intermetallic compounds (IMCs) in solder joints after long-term high-temperature operation is a crucial factor for the reliability of SB solders. Growing IMC layers can degrade the overall strength and lifetime of solder joints due to their brittle nature. Hence, a thermal aging test was conducted by placing the as-bonded solder joints into a temperature-controllable oven at 100 °C for 100 h. To minimize the effect of the coefficient of thermal expansion (CTE) mismatch within the solder joints, a very slow heating process was applied (~ 1 °C/min) before reaching the test temperature of (100 °C).

### Thermal cycle test

As mentioned in the previous section, overheating or operating under extreme environmental conditions can cause severe degradation of solder joints, leading to permanent damage to optical and electrical devices. Therefore, thermal cycle tests were conducted on the studied solder joints. Accelerated thermal cycling within a wide temperature window of 0 to 100 °C) was used. During each cycle, the temperature was ramped up from 0 to 100 °C in 15 min, dwelt at 100 °C for 30 min, ramped down to 0 °C in 15 min, and finally dwelt at 0 °C for 30 min [30]. A temperature humidity test chamber (Model: LRHS-101-LH, Shanghai Linpin Instrument Stock Co., LTD.) was used for the thermal cycling tests.

### Differential scanning calorimetry (DSC) measurement

The melting point of the fabricated solder sheets was measured using a differential scanning calorimetry (DSC) (DSC 404 F1, NETZSCH). During the measurements, a small piece of solder sheet was placed in a sapphire crucible that underwent the measurements in a N_2_ gas environment with a heating rate 20 K/min. At each RE concentration, the DSC data were collected on three samples, and each sample was measured three times. The average value for each RE concentration was recorded as the melting point.

## Results and discussion

### Characterization of RE-containing Sn–Bi solder

**Fig. 4:**
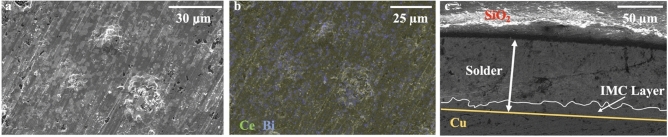
Microstructure of solder sheets and Cu/SnBi-6RE/SiO_2_ solder joints. **a** SEM image of an as-fabricated SnBi-6RE solder sheet. **b** Corresponding EDX mapping of Ce (yellow) and Bi (blue) elements in the SnBi-6RE solder sheet. **c** SEM image of cross-section view of as-bonded Cu/SnBi-6RE/SiO_2_ solder joints (Color figure online)

To characterize our solder sheets, we conducted scanning electron microscopy (SEM) imaging and energy-dispersive X-ray analysis (EDAX) analysis (FEI Quanta FEG250). The results are shown in Fig. 4. The microstructure of our RE solders was consistent with previous research [5, 11]. From the EDAX data, we found that the doped RE elements were evenly distributed within the solder sheet. Additionally, we investigated the weight percentages of each element in three different locations using EDX. For the sample with 6 wt% RE, the average RE concentration across these locations was found to be 6.6%, showing successful doping of the RE elements with the expected concentration. Based on these findings, we concluded that our fabrication process can lead to consistent production of the desired universal solder sheets.

In Fig. 4c, the cross-section view of the solder joints shows a solder layer thickness of less than 100 μm. At the interface between copper and solder, a non-flat intermetallic layer (IML) was observed which is consistent with previous studies [26–28]. The thickness of the IML was typically less than 10 μm according to the SEM images.

**Fig. 5:**
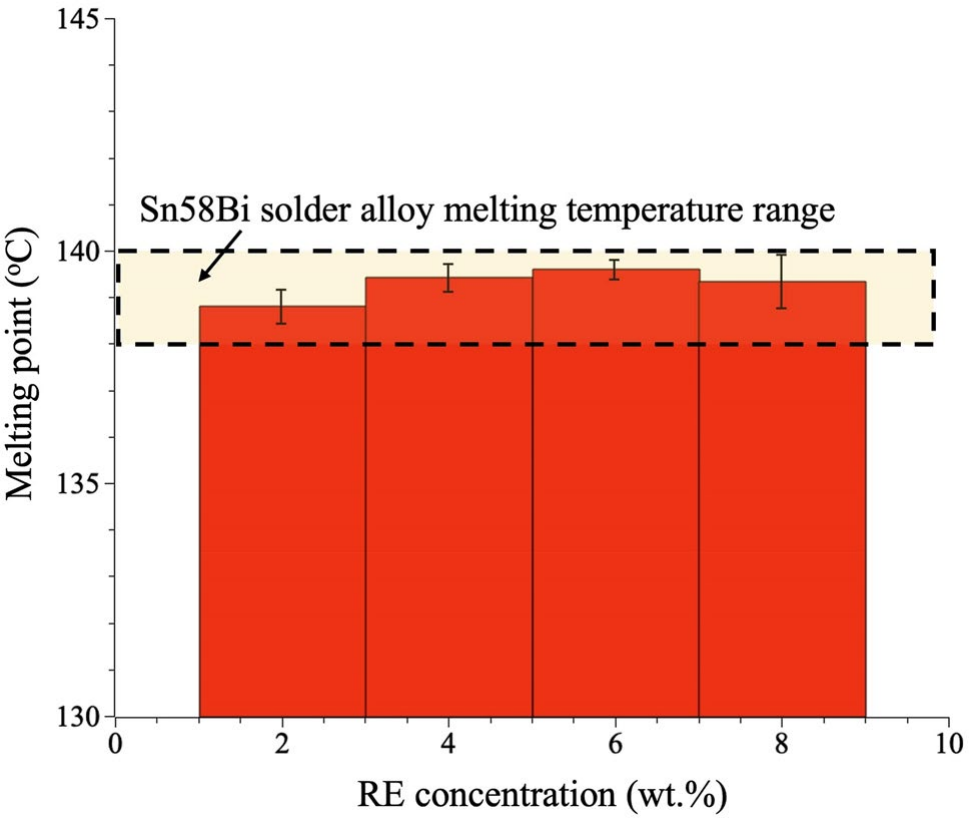
Measured melting points of as-fabricated solder sheets with different RE concentrations

Prior to optimizing the soldering process, we used differential scanning calorimetry to measure the melting point as a function of RE concentration, as shown in Fig. 5. Our results indicated that adding trace amounts of RE to the original solder matrix (Sn–58Bi) does not alter its original melting point (138–140 °C), which is consistent with previous findings [5].

#### Solder joints shear strength under different soldering conditions

**Fig. 6:**
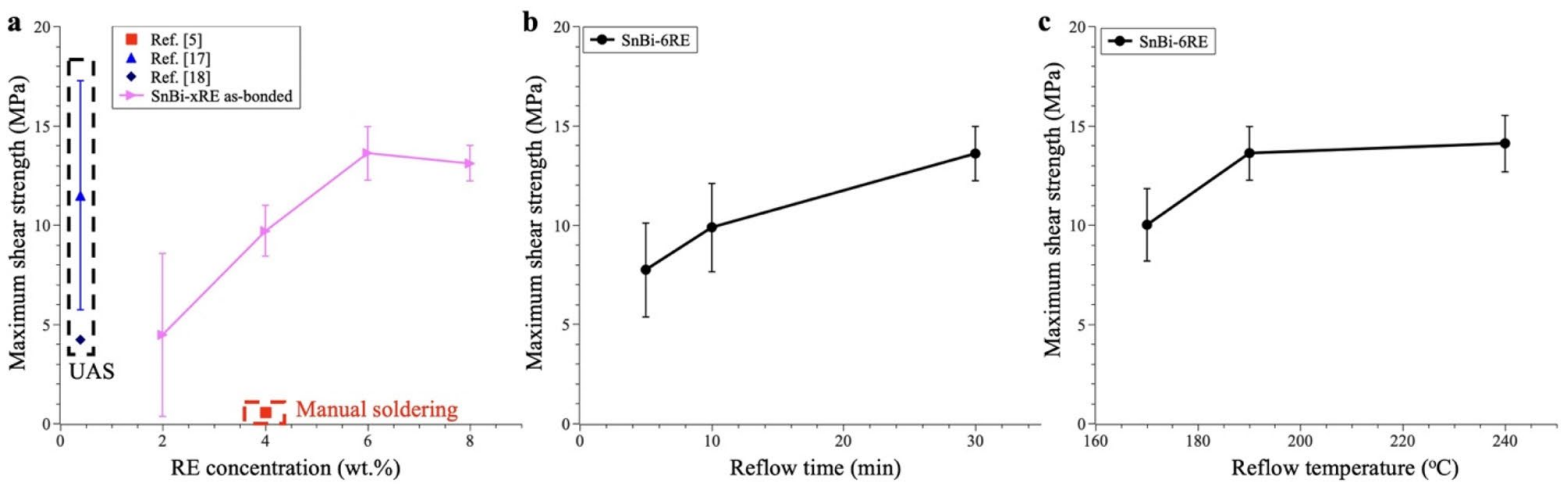
Maximum shear strength of Cu/SnBi-xRE/SiO_2_ solder joints fabricated under different conditions. **a** Effect of RE concentrations on the maximum shear strength of as-bonded solder joints. The reflow conditions were 190 °C, 30 min, in Ar. **b** Effect of reflow time on solder joint strength. The RE concentration was fixed to be 6% (SnBi-6RE). The reflow temperature was 190 °C. **c** Effect of reflow temperature on the solder joint strength. The RE concentration was fixed to be 6% (SnBi-6RE). The reflow time was 30 min

Figure 6 compares the maximum shear strength of copper-glass solder joints under different soldering conditions in an Ar atmosphere, as described in the previous section. Each data point represents the average results from 6 to 8 samples, with the error bar indicating the standard deviation. We first studied four different weight percentages of RE concentration: 2%, 4%, 6%, and 8%, as shown in Fig. 6a. It was evident that 6% and 8% RE concentrations exhibited higher shear strength compared to the ones with the lower RE concentrations, with a slightly higher average shear strength observed with the 6% RE concentration. Therefore, we selected the 6% RE solder sheets as the optimized concentration. Figure 6a also compares our results with the reported solders using similar active Bi–Sn solder mixtures on bare glass substrates, including bonding with an ultrasonic-assisted soldering (UAS) process and a manual process. Compared to these results, our universal solder joints bonded with the process developed in this study show the highest shear strength.

Figure 6b shows the impact of the reflow time on the solder joint strength. Increasing the reflow time from 5 to 30 min resulted in a higher average maximum shear strength. This can be attributed to longer reaction and diffusion times, enabling better bonding at the interface. Similar trends have been observed in the maximum strength of Cu/SnAgTi/SiO_2_ solder joints with increasing soldering time [22]. The bonding nature of the universal solders is induced by the high oxygen affinity of active elements, like Ti, Zn, and RE. During the bonding process, oxygen atoms in the ceramics or glass form bonds with these active elements at the oxidized interface (e.g., SiO_2_ in this study) through diffusion effects [11–13, 17, 18]. Sufficient reflow time is beneficial to allow active elements to diffuse to the interface for optimal bonding.

We further investigated the effect of reflow temperature on the solder joint strength by selecting three different soldering temperatures. Figure 6(c) shows that there was no significant increase in solder joint strength above a reflow temperature of 190 °C. Based on these results, we optimized the soldering process with a 6% RE solder sheet, a 30-minute reflow time at 190 °C, and an applied pressure of 0.2 MPa. This optimized procedure achieved an average maximum shear strength of 13.6 MPa in copper-glass solder joints, surpassing all the previously reported values on similar active SB solders using either UAS or manual soldering procedures. Compared with the state-of-art active solder joints summarized in Table 1, our optimized soldering process achieved the highest strength amongst all the similar SB solders, and possessed comparable strength to SnAg-based active solders, which have a much higher melting point (~ 221 °C). Therefore, the combination of the high shear strength and a low melting point of our RE-doped SB solder is promising for applications in temperature-sensitive optoelectronic devices.

#### Solder joints stability during thermal aging and cycling

As mentioned earlier, both thermal aging and thermal cycling have significant impacts on the solder joint reliability. At elevated temperatures, the solder matrix undergoes coarsening, and the IML thickness at the copper-solder interface increases with prolonged device exposure. These effects contribute to the degradation of solder joint strength [2, 13, 27–29]. Additionally, the coefficient of thermal expansion (CTE) mismatch between ceramic/oxide substrates (e.g., SiO_2_ with a CTE of ~ 5 × 10^−6^ K^−1^) and metal substrates (e.g., Cu with a CTE of ~ 17 × 10^−6^ K^−1^) also leads to thermal stress that can cause permanent damage to electronic devices [25, 30–34].

**Fig. 7:**
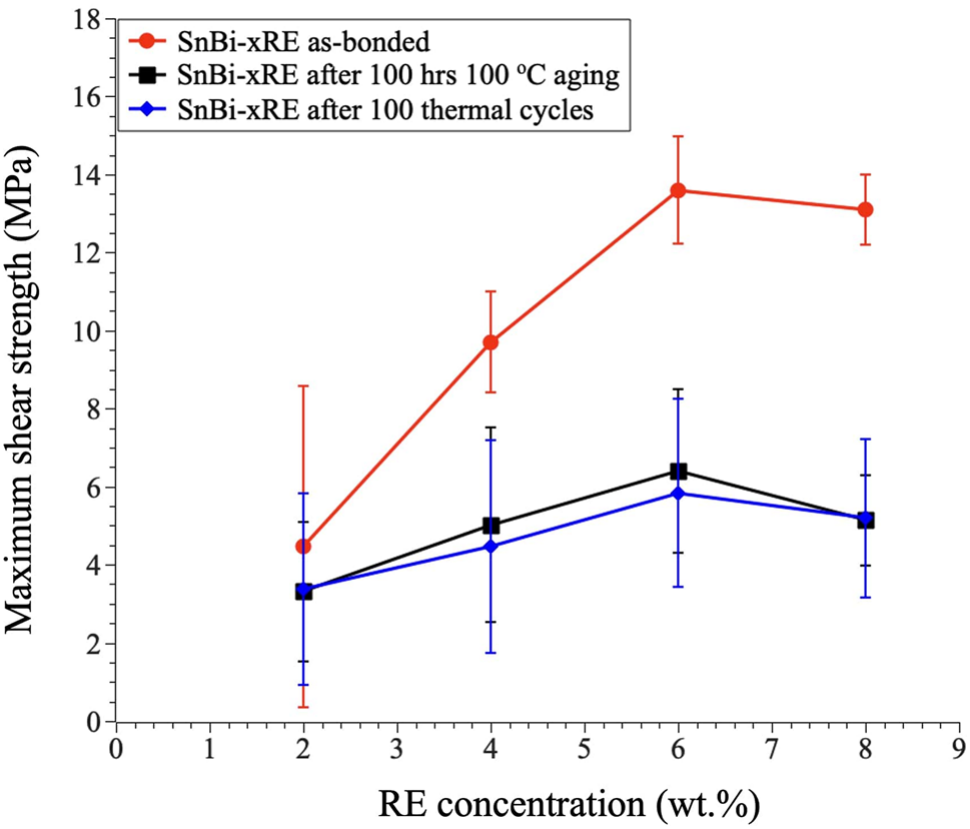
Impact of thermal aging and cycling on the maximum shear strength of Cu/SnBi-xRE/SiO_2_ solder joints. All the joints were made with reflow temperature at 190  for 30 min in Ar )

In this study, we investigated the effects of thermal aging and thermal cycling on the Cu/SnBi-xRE/SiO_2_ solder joints. Comparing with the results with as-bonded solder joints, both thermal processes resulted in a decrease in the maximum shear strength of the solder joints, with slightly larger degradation observed after the thermal cycling test as shown in Fig. 7. Interestingly, while the solder sheets with 6% RE concentration exhibited the highest shear strength among different RE concentrations in the as-bonded joints, the shear strength diminished after the thermal processes, suggesting that the benefit of larger strength at the glass-solder interface with higher RE concentration is diminished with thermal aging and cycling.

**Fig. 8:**
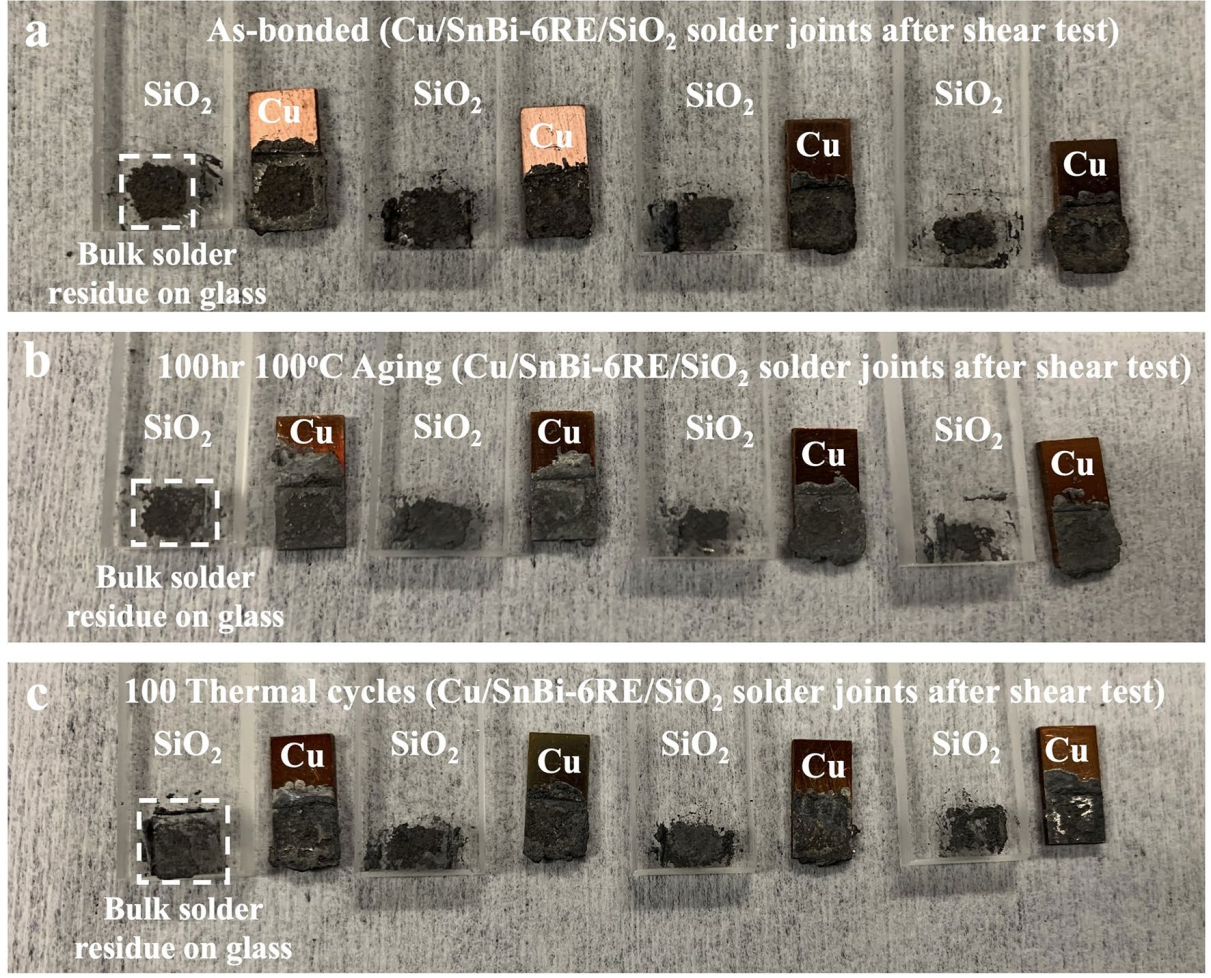
Photographs of Cu/SnBi-6RE/SiO_2_ solder joints after shear tests with and without post-thermal processes. **a** as-bonded without thermal process. **b** after 100-hr 100 °C thermal aging. **c** after 100 thermal cycles between 0 and 100 °C. In (**a–c**), the highlighted regions indicate large amounts of bulk solder left on the glass side after the shear tests

**Fig. 9:**
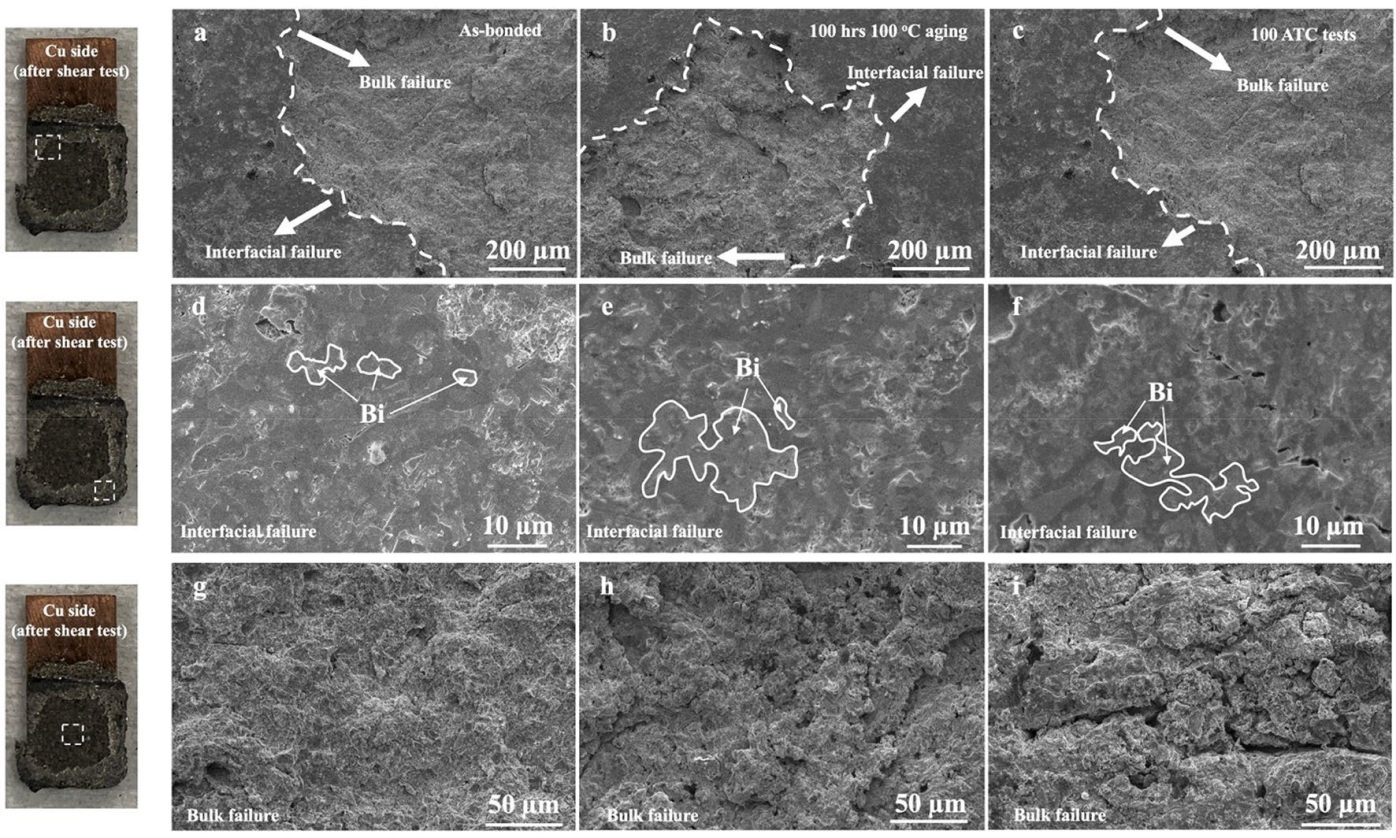
SEM images of the Cu/SnBi-6RE/SiO_2_ solder joints after the shear tests on samples before and after the post-thermal processes. All the images were taken from the top surfaces of the solders that remained on Cu substrates after the shear tests. **a–c**: SEM images with both interfacial and bulk failure regions; **d–f** SEM images of interfacial failure regions; **g–i** SEM images of bulk failure regions; (**a**) (**d**) (**g**) were different failure locations from as-bonded solder joints; (**b**) (**e**) (**h**) were different failure locations of solder joints after thermal aging; (**c**) (**f**) (**i**) were different failure locations of solder joints after thermal cycles

To better understand the impact of thermal processing on solder joint strength, we investigated the microstructures of the solder joints on the fractured solder surface after the shear tests. First, we examined the failure structure of the as-bonded solder joints prior to thermal processing. After the shear test, a larger amount of solder remained on the glass and Cu substrates, indicating that failure predominantly occurred within the bulk solder itself rather than at the glass-solder or Cu-solder interface, as shown in Fig. 8. The failure types can be classified as a combination of ‘interfacial’ and ‘bulk region’ failures [18]. Further examination of the microstructure of the fractured solder joints through SEM imaging revealed that all three types of conditions (as-bonded, after aging, after cycling) exhibited both bulk and interfacial failure modes (Fig. 9). Moreover, we observed a coarsened microstructure and increased Bi-phase area after the thermal aging and cycling, presumably due to the thermal effect on the solder alloy [2, 13, 27]. Within the bulk failure region, we observed different morphologies before and after the thermal processes. Comparing the images, we found more discrete small shapes after thermal aging, which may be attributed to the brittleness of IMC layer. The thermal stress induced by the CTE mismatch could cause crack propagation, leading to larger cracks and voids, as observed after thermal cycling. Similar cracks were also present in the interfacial failure region after the thermal cycle test. Therefore, we conclude that the lower shear strength after thermal cycling is a result of thermal stress-induced cracks within the bulk solder due to CTE mismatch.

**Fig. 10:**
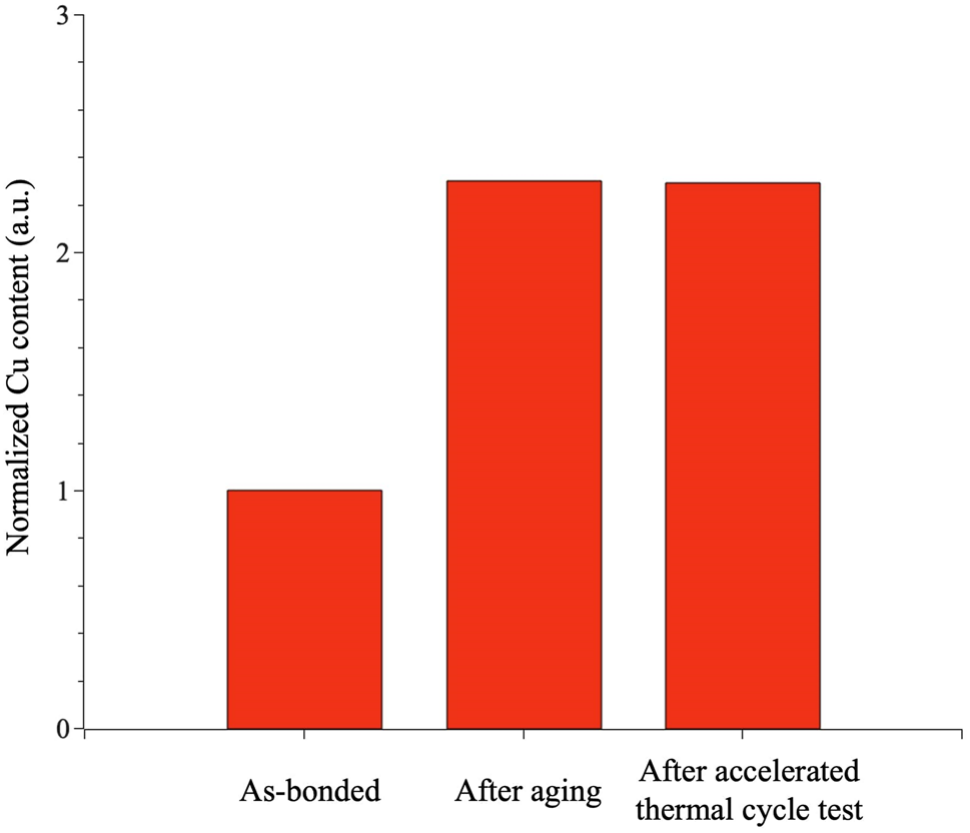
Cu content in the bulk failure regions from the fractured Cu/SnBi-6RE/Glass solder joints after shear tests. Results are normalized by the averaged Cu contents in the as-bonded samples

To further support this hypothesis, we analyzed the Cu concentration within the bulk failure regions under the three test conditions. Cu elements were initially introduced during the soldering process, forming an IMC layer at the interface with the solder. The thickness of this layer then could grow during the post-thermal processes, resulting in weaker joint strength. Since IMC is more brittle than the bulk solder, it is more prone to break during shear tests. Indeed, we observed higher Cu concentration after the thermal processes compared to the as-bonded solder joints (Fig. 10), suggesting the growth of the IMC layer at elevated temperatures that caused the lower solder joint strength.

## Conclusion

In summary, we have demonstrated that doping a trace amount of rare earth elements (mischmetal Ce:La 75:25 wt%) into the conventional Sn–58Bi (SB) solder matrix enables direct and strong bonding to oxide substrates without the need for metallization treatment. Through our optimized soldering process under Ar atmosphere, the highest average shear strength of 13.6 MPa was obtained based on a Cu/ SnBi-6RE /SiO_2_ configuration (i.e., with 6 wt% of RE in the SB matrix). The soldering process is free of ultrasonic assistance and is done at a relatively low soldering temperature (~ 190 °C). To assess the thermal stability of the Cu/SnBi-xRE/SiO_2_ solder joints, we conducted both thermal aging and cycling tests. We observed degradation in the maximum shear strength after both post-thermal processes, with slightly larger degradation observed after the thermal cycling test. Coarsened microstructures were also observed after exposure to elevated temperatures in both tests. Furthermore, larger voids and cracks were observed in failed solder joints after the thermal cycle test, likely due to CTE mismatches within the solder joints. Based on our findings, we conclude that the main factor contributing to the strength degradation of the joints is the bulk region of the solder after thermal treatments, rather than the interfacial bonding between SiO_2_ and the solder. This study highlights that the optimized soldering process with RE-doped SB solders yields the highest shear strength results for Cu-glass solder joints compared to the undoped SB solders. Our investigation provides a potential solution for direct bonding on optoelectronic substrates without surface metallization, utilizing a low-temperature soldering process. To further enhance the thermal stability of the bonded devices, particularly in terms of bulk solder strength improvements after thermal treatments, further work is warranted on improving the solder strength.

## Data Availability

The data that support the findings of this study are available within the paper. Other relevant data are available from the corresponding authors on reasonable request.
